# Poly[[diaqua­deca-μ-cyanido-hexa­cyanidobis­(4-cyano­pyridine)di-μ-pyrimidine-tricopper(II)ditungsten(V)] dihydrate]

**DOI:** 10.1107/S1600536808032947

**Published:** 2008-10-18

**Authors:** Souhei Kaneko, Yoshihide Tsunobuchi, Koji Nakabayashi, Shin-ichi Ohkoshi

**Affiliations:** a Department of Chemistry, School of Science, University of Tokyo, 7-3-1 Hongo, Bunkyo-Ku, Tokyo 113-0033, Japan

## Abstract

In the polymeric title compound, {[Cu_3_W_2_(CN)_16_(C_4_H_4_N_2_)_2_(C_6_H_4_N_2_)_2_(H_2_O)_2_]·2H_2_O}_*n*_, the coordination geometry of W is an eight-coordinated bicapped trigonal prism. Five of the CN groups of [W(CN)_8_] are bridged to Cu ions. The coordination geometries of the Cu atoms are each pseudo-octa­hedral; one Cu atom is located on a centre of inversion. The cyano-bridged W–Cu layers are linked by Cu-containing pillars, to form a three-dimensional network with cavities occupied by noncoordinated water and 4-cyano­pyridine mol­ecules.

## Related literature

For general background, see: Arimoto *et al.* (2003 ▶); Catala *et al.* (2005 ▶); Hozumi *et al.* (2003 ▶); Leipoldt *et al.* (1994 ▶); Ohkoshi *et al.* (2006 ▶, 2008 ▶); Pilkington & Decurtins (2000 ▶); Zhong *et al.* (2000 ▶). For related structures, see: Garde *et al.* (1999 ▶); Ohkoshi *et al.* (2003 ▶, 2007 ▶).
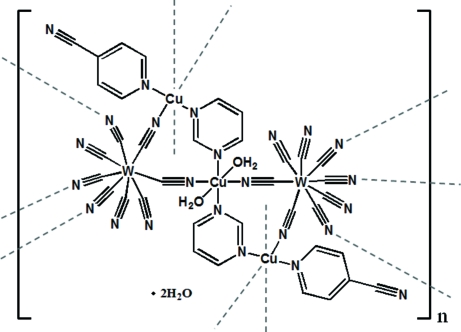


## Experimental

### 

#### Crystal data

[Cu_3_W_2_(CN)_16_(C_4_H_4_N_2_)_2_(C_6_H_4_N_2_)_2_(H_2_O)_2_]·2H_2_O
*M* *_r_* = 1407.02
Monoclinic, 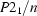

*a* = 7.2475 (6) Å
*b* = 15.4532 (12) Å
*c* = 20.8560 (16) Å
β = 90.057 (2)°
*V* = 2335.8 (3) Å^3^
*Z* = 2
Mo *K*α radiation
μ = 6.32 mm^−1^
*T* = 90 (2) K
0.44 × 0.17 × 0.04 mm

#### Data collection

Rigaku R-AXIS RAPID diffractometer
Absorption correction: numerical (*ABSCOR*; Higashi, 1995 ▶) *T*_min_ = 0.297, *T*_max_ = 0.791
22562 measured reflections
5345 independent reflections
4975 reflections with *I* > 2σ(*I*)
*R*_int_ = 0.095

#### Refinement

*R*[*F* ^2^ > 2σ(*F* ^2^)] = 0.039
*wR*(*F* ^2^) = 0.093
*S* = 1.09
5345 reflections
314 parameters
H-atom parameters constrained
Δρ_max_ = 2.97 e Å^−3^
Δρ_min_ = −1.45 e Å^−3^

### 

Data collection: *PROCESS-AUTO* (Rigaku, 1998 ▶); cell refinement: *PROCESS-AUTO*; data reduction: *CrystalStructure* (Rigaku Americas & Rigaku, 2007 ▶); program(s) used to solve structure: *SHELXS97* (Sheldrick, 2008 ▶); program(s) used to refine structure: *SHELXL97* (Sheldrick, 2008 ▶); molecular graphics: *ORTEP-3* (Farrugia, 1997 ▶) and *PyMOLWin* (DeLano, 2007 ▶); software used to prepare material for publication: *CrystalStructure*.

## Supplementary Material

Crystal structure: contains datablocks global, I. DOI: 10.1107/S1600536808032947/tk2308sup1.cif

Structure factors: contains datablocks I. DOI: 10.1107/S1600536808032947/tk2308Isup2.hkl

Additional supplementary materials:  crystallographic information; 3D view; checkCIF report

## Figures and Tables

**Fig. 1.**
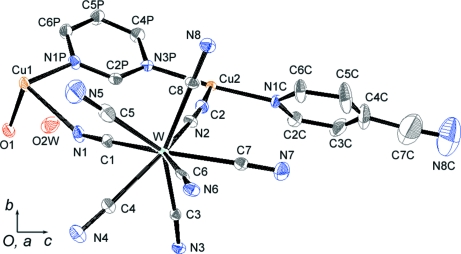
Displacement ellipsoid plot (50% probability level) of (I) showing the asymmetric unit. Hydrogen atoms are omitted for clarity.

**Fig. 2.**
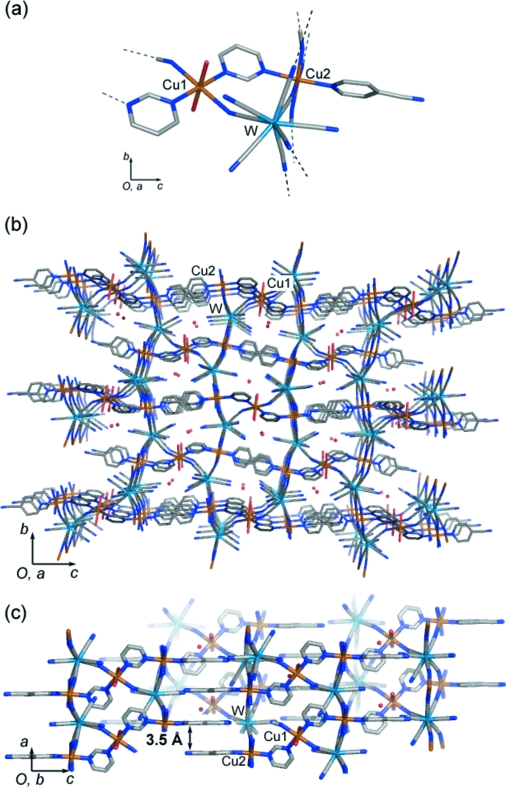
Supramolecular connectivity in (I) where hydrogen atoms are omitted for clarity. (a) The coordination environment around the W and Cu atoms. The broken lines indicate coordination to symmetry-related metal ions. (b) View along the a axis, the direction of the formed pores. (c) View along the b axis. Colour code: Light blue, orange, gray, blue, and red represent W, Cu, C, N, and O atoms, respectively.

## supplementary crystallographic information

### Comment

The preparation of ferromagnetic nanoporous materials is an attractive contemporary research area. An octacyanometalate [*M*(CN)_8_] (*M* = Mo, W, Nb)-based magnets are good candidates because of their high Curie temperatures (Garde *et al.*, 1999; Zhong *et al.*, 2000; Pilkington & Decurtins, 2000), functionalities such as photomagnetism (Arimoto *et al.*, 2003; Catala *et al.*, 2005; Ohkoshi *et al.*, 2006,2008) and chemically sensitive magnetism (Ohkoshi *et al.*, 2007). Octacyanometalates, [*M*(CN)_8_]^n-^, a versatile class of building blocks, can adopt different spatial configurations depending on the coordinating ligands, *e.g.*, square antiprismic (D_4h_), dodecahedral (D_2d_), and bicapped trigonal prismic (C_2v_) (Leipoldt *et al.*, 1994). In the case of Cu—W systems, several octacyanometalate-based magnets such as {[Cu_3_[W(CN)_8_]_2_]3.4H_2_O}_n_ (3-dimensional network complex, 3-D) (Garde *et al.*, 1999), {[Cu_3_[W(CN)_8_]_2_(pyrimidine)_2_]8H_2_O}_n_ (3-D) (Ohkoshi *et al.*, 2007), {[Cu_3_[W(CN)_8_]_2_(3-cyanopyridine)_6_]4H_2_O}_n_ (2-D array), and {[Cu_3_[W(CN)_8_]_2_(4-cyanopyridine)_6_]8H_2_O}_n_ (2-D array) (Ohkoshi *et al.*, 2003), have been reported.

The asymmetric unit of the present compound (I) comprises a [W(CN)_8_]^3-^ anion, a one-half of [Cu1(H_2_O)_2_]^2+^ cation (the Cu centre is located on a centre of inversion), a [Cu_2_(pyrimidine)(4-cyanopyridine)]^2+^ cation, and a water molecule, Fig. 1. The coordination geometry of W is eight-coordinated bicapped trigonal prismic, where five CN groups of [W(CN)_8_] are bridged to Cu ions (one Cu1 and four Cu2), and the other three CN groups are free (Fig. 2a). The coordination geometries of the two types of Cu^II^ ions (Cu1 and Cu2) are pseudo-octahedral. The Cu1 atom is coordinated to two N atoms of CN ligands, two N atoms of pyrimidine molecules, and two O atoms of H_2_O molecules. The Cu2 atom is coordinated to four N atoms of CN ligands, one N atom of a pyrimidine molecule, and one N atom of a 4-cyanopyridine molecule.

The cyano-bridged-Cu2—W layers are linked by Cu1 pillar unit (Figs 2b and 2c). This arrangement leads to the formation of cavities along *a* axis which are occupied by 4-cyanopyridine molecules and zeolitic-like water molecules (Fig. 2b). The 4-cyanopyridine molecules are aligned alternately without forming significant intermolecular interaction, Fig. 2c.

The field-cooled magnetization (FCM) curve at 10 Oe showed a spontaneous magnetization with a Curie temperature (*T*_c_) of 12 K, the coercive field (*H*_c_) of 70 Oe at 2 K, and, the saturation magnetization (*M*_s_) value of 3.1 *µ*_B_. This *M*_s_ value indicates that this compound is a ferrimagnet in which W^V^ (S = 1/2) and Cu^II^ (S = 1/2, Cu2) in the layer are ferromagnetically coupled and W^V^ and the bridged Cu^II^ (S = 1/2, Cu1) are antiferromagnetically coupled.

### Experimental

The title compound was prepared by reacting an aqueous solution of Cs_3_[W(CN)_8_]2H_2_O (1.2 × 10 ^-2^ mol dm^-3^) with a mixed aqueous solution of CuCl_2_.2H_2_O (1.8 × 10 ^-2^ mol dm^-3^), 4-cyanopyridine (1.8 × 10 ^-2^ mol dm^-3^) and pyrimidine (1.2 × 10 ^-2^ mol dm^-3^) at room temperature. The prepared compound was a green plate-like crystal. Elemental analysis found: C 30.36, H 1.88, N 23.99, Cu 13.84, W 26.20; C_36_H_24_N_24_O_4_Cu_3_W_2_ requires: Cu, 13.47; W, 25.98; C, 30.56; H, 1.71; N, 23.76.

In the IR spectrum, cyano stretching peaks were observed at 2154, 2162, 2170, and 2200 cm^-1^. The UV-visible reflectance spectrum showed absorption bands at around 700 and 1070 nm.

### Refinement

The H atoms of the solvent water molecules and the coordinated water molecules could not be located reliably and were not included in the refinement. The remaining H atoms were placed in calculated positions and refined using a riding model, with C-H = 0.95 Å, and with *U*_iso_(H) = 1.2 *U*_eq_(C). The maximum and minimum residual electron density peaks were located 0.66 and 1.61 Å, respectively from the W atom.

### Crystal data

**Table:**

[Cu_3_W_2_(CN)_16_(C_4_H_4_N_2_)_2_(C_6_H_4_N_2_)_2_(H_2_O)_2_]·2H_2_O	*F*(000) = 1334
*M**_r_* = 1407.02	*D*_x_ = 2.000 Mg m^−^^3^
Monoclinic, *P*2_1_/*n*	Mo *K*α radiation, λ = 0.71075 Å
Hall symbol: -P 2yn	Cell parameters from 15466 reflections
*a* = 7.2475 (6) Å	θ = 3.1–27.5°
*b* = 15.4532 (12) Å	µ = 6.32 mm^−^^1^
*c* = 20.8560 (16) Å	*T* = 90 K
β = 90.057 (2)°	Plate, green
*V* = 2335.8 (3) Å^3^	0.44 × 0.17 × 0.04 mm
*Z* = 2	

### Data collection

**Table:**

Rigaku R-AXIS RAPID diffractometer	5345 independent reflections
Radiation source: sealed tube	4975 reflections with *I* > 2σ(*I*)
sealed tube	*R*_int_ = 0.095
Detector resolution: 10.00 pixels mm^-1^	θ_max_ = 27.5°, θ_min_ = 3.1°
ω scans	*h* = −9→9
Absorption correction: numerical (ABSCOR; Higashi, 1995)	*k* = −20→17
*T*_min_ = 0.297, *T*_max_ = 0.791	*l* = −27→26
22562 measured reflections	

### Refinement

**Table:**

Refinement on *F*^2^	0 constraints
Least-squares matrix: full	Secondary atom site location: structure-invariant direct methods
*R*[*F*^2^ > 2σ(*F*^2^)] = 0.039	Hydrogen site location: inferred from neighbouring sites
*wR*(*F*^2^) = 0.093	H-atom parameters constrained
*S* = 1.09	*w* = 1/[σ^2^(*F*_o_^2^) + 6.8782*P*] where *P* = (*F*_o_^2^ + 2*F*_c_^2^)/3
5345 reflections	(Δ/σ)_max_ = 0.002
314 parameters	Δρ_max_ = 2.97 e Å^−^^3^
0 restraints	Δρ_min_ = −1.45 e Å^−^^3^

### Special details

**Table:**

Refinement. Refinement was performed using all reflections. The weighted *R*-factor (*wR*) and goodness of fit (*S*) are based on *F*^2^. *R*-factor (gt) are based on *F*. The threshold expression of *F*^2^ > 2.0 σ(*F*^2^) is used only for calculating *R*-factor (gt).

### Fractional atomic coordinates and isotropic or equivalent isotropic displacement parameters (Å^2^)

**Table:**

	*x*	*y*	*z*	*U*_iso_*/*U*_eq_	
W(1)	0.79222 (2)	0.682386 (11)	0.301091 (8)	0.00933 (7)	
Cu(1)	0.5000	0.5000	0.5000	0.01726 (18)	
Cu(2)	0.24342 (7)	0.43738 (4)	0.23347 (3)	0.01074 (13)	
O(1)	0.6198 (4)	0.3863 (2)	0.50585 (15)	0.0199 (7)	
O(2W)	0.4469 (5)	0.2609 (2)	0.44669 (18)	0.0331 (9)	
N(8)	0.4520 (5)	0.8223 (2)	0.2839 (2)	0.0171 (8)	
N(5)	0.7372 (8)	0.7918 (4)	0.4362 (2)	0.0445 (14)	
N(4)	1.1739 (5)	0.6555 (3)	0.3867 (2)	0.0221 (9)	
N(6)	1.0499 (5)	0.8542 (2)	0.27039 (19)	0.0155 (8)	
N(3P)	0.2076 (5)	0.4190 (2)	0.33127 (19)	0.0139 (7)	
N(3)	1.0395 (5)	0.5230 (2)	0.24087 (18)	0.0146 (8)	
N(7)	0.7775 (6)	0.7133 (3)	0.1440 (2)	0.0251 (9)	
N(1P)	0.3037 (5)	0.4489 (2)	0.43733 (19)	0.0167 (8)	
N(1C)	0.2581 (5)	0.4584 (2)	0.13674 (18)	0.0160 (8)	
N(2)	0.4536 (5)	0.5548 (2)	0.25142 (19)	0.0159 (8)	
N(8C)	0.2655 (15)	0.5073 (7)	−0.1191 (3)	0.108 (3)	
N(1)	0.6858 (5)	0.5382 (3)	0.41107 (19)	0.0195 (9)	
C(5)	0.7573 (7)	0.7547 (3)	0.3890 (2)	0.0247 (11)	
C(8)	0.5679 (6)	0.7718 (3)	0.2897 (2)	0.0131 (8)	
C(6)	0.9565 (6)	0.7950 (3)	0.2801 (2)	0.0149 (9)	
C(1)	0.7250 (6)	0.5869 (3)	0.3723 (2)	0.0151 (9)	
C(3)	0.9483 (5)	0.5780 (3)	0.2595 (2)	0.0117 (8)	
C(2)	0.5694 (6)	0.6008 (3)	0.2667 (2)	0.0139 (9)	
C(7)	0.7801 (6)	0.7033 (3)	0.1982 (2)	0.0147 (9)	
C(4)	1.0445 (6)	0.6656 (3)	0.3562 (2)	0.0145 (9)	
C(4P)	0.0484 (6)	0.3870 (3)	0.3537 (2)	0.0208 (10)	
C(5P)	0.0114 (6)	0.3841 (4)	0.4186 (2)	0.0250 (11)	
C(2P)	0.3299 (6)	0.4475 (3)	0.3738 (2)	0.0158 (9)	
C(6C)	0.2623 (8)	0.5390 (3)	0.1143 (2)	0.0286 (12)	
C(5C)	0.2606 (10)	0.5585 (4)	0.0505 (3)	0.0468 (19)	
C(4C)	0.2565 (9)	0.4902 (6)	0.0079 (3)	0.052 (2)	
C(3C)	0.2549 (8)	0.4065 (5)	0.0295 (2)	0.0412 (17)	
C(2C)	0.2567 (7)	0.3938 (3)	0.0953 (2)	0.0252 (11)	
C(6P)	0.1443 (6)	0.4167 (4)	0.4596 (2)	0.0238 (11)	
C(7C)	0.2648 (14)	0.4950 (8)	−0.0622 (4)	0.083 (3)	
H(4P)	−0.0415	0.3657	0.3239	0.025*	
H(5P)	−0.1017	0.3605	0.4346	0.030*	
H(2P)	0.4451	0.4683	0.3579	0.019*	
H(6C)	0.2664	0.5859	0.1449	0.035*	
H(5C)	0.2618	0.6178	0.0358	0.056*	
H(3C)	0.2521	0.3584	−0.0000	0.050*	
H(2C)	0.2567	0.3355	0.1117	0.031*	
H(6P)	0.1222	0.4165	0.5050	0.028*	

### Atomic displacement parameters (Å^2^)

**Table:**

	*U*^11^	*U*^22^	*U*^33^	*U*^12^	*U*^13^	*U*^23^
W(1)	0.00709 (11)	0.00851 (13)	0.01238 (11)	−0.00006 (5)	−0.00063 (7)	0.00014 (6)
Cu(1)	0.0109 (3)	0.0285 (5)	0.0123 (3)	−0.0043 (3)	−0.0029 (2)	0.0043 (3)
Cu(2)	0.0096 (2)	0.0105 (2)	0.0121 (2)	0.00227 (19)	−0.00008 (18)	0.0000 (2)
O(1)	0.0141 (16)	0.029 (2)	0.0161 (15)	−0.0006 (13)	−0.0023 (11)	0.0056 (15)
O(2W)	0.029 (2)	0.040 (2)	0.030 (2)	−0.0009 (17)	0.0017 (16)	−0.015 (2)
N(8)	0.016 (2)	0.015 (2)	0.020 (2)	0.0002 (14)	0.0004 (15)	0.0026 (16)
N(5)	0.056 (3)	0.049 (3)	0.029 (2)	0.017 (2)	−0.008 (2)	−0.020 (2)
N(4)	0.019 (2)	0.022 (2)	0.025 (2)	−0.0013 (17)	−0.0066 (16)	0.005 (2)
N(6)	0.0106 (18)	0.015 (2)	0.021 (2)	−0.0015 (15)	−0.0004 (14)	0.0014 (17)
N(3P)	0.0123 (18)	0.013 (2)	0.0160 (18)	0.0003 (14)	0.0009 (13)	0.0001 (16)
N(3)	0.0109 (18)	0.015 (2)	0.0175 (18)	0.0003 (15)	0.0000 (13)	0.0012 (16)
N(7)	0.029 (2)	0.026 (2)	0.021 (2)	0.0002 (19)	0.0035 (16)	0.006 (2)
N(1P)	0.0151 (19)	0.018 (2)	0.0169 (19)	−0.0028 (15)	−0.0023 (14)	0.0050 (17)
N(1C)	0.0145 (19)	0.022 (2)	0.0113 (18)	−0.0018 (15)	0.0007 (13)	0.0037 (17)
N(2)	0.0154 (19)	0.016 (2)	0.0160 (18)	−0.0005 (15)	−0.0012 (14)	−0.0003 (16)
N(8C)	0.145 (9)	0.136 (10)	0.043 (4)	−0.015 (7)	−0.005 (4)	−0.003 (5)
N(1)	0.016 (2)	0.026 (2)	0.0161 (19)	−0.0067 (16)	−0.0049 (14)	0.0059 (19)
C(5)	0.025 (2)	0.023 (2)	0.027 (2)	0.004 (2)	−0.0081 (19)	−0.001 (2)
C(8)	0.012 (2)	0.012 (2)	0.015 (2)	0.0016 (16)	−0.0008 (15)	−0.0019 (18)
C(6)	0.010 (2)	0.016 (2)	0.018 (2)	0.0041 (18)	−0.0037 (15)	0.004 (2)
C(1)	0.012 (2)	0.021 (2)	0.013 (2)	−0.0017 (17)	−0.0036 (15)	−0.003 (2)
C(3)	0.011 (2)	0.014 (2)	0.0102 (19)	−0.0022 (17)	−0.0015 (14)	−0.0006 (18)
C(2)	0.011 (2)	0.012 (2)	0.018 (2)	−0.0003 (17)	−0.0018 (15)	0.0016 (19)
C(7)	0.011 (2)	0.014 (2)	0.019 (2)	−0.0024 (17)	0.0012 (15)	0.003 (2)
C(4)	0.014 (2)	0.013 (2)	0.017 (2)	−0.0028 (17)	−0.0022 (16)	0.0022 (19)
C(4P)	0.013 (2)	0.028 (2)	0.022 (2)	−0.0076 (19)	−0.0006 (17)	−0.002 (2)
C(5P)	0.016 (2)	0.039 (3)	0.020 (2)	−0.007 (2)	0.0037 (17)	0.002 (2)
C(2P)	0.012 (2)	0.017 (2)	0.018 (2)	0.0001 (17)	0.0005 (16)	−0.0006 (19)
C(6C)	0.042 (3)	0.024 (2)	0.020 (2)	−0.012 (2)	−0.007 (2)	0.010 (2)
C(5C)	0.061 (4)	0.047 (4)	0.032 (3)	−0.032 (3)	−0.018 (2)	0.028 (3)
C(4C)	0.032 (3)	0.100 (6)	0.024 (3)	−0.024 (3)	−0.008 (2)	0.028 (3)
C(3C)	0.034 (3)	0.070 (5)	0.020 (2)	−0.001 (3)	0.005 (2)	−0.012 (3)
C(2C)	0.027 (2)	0.029 (3)	0.020 (2)	0.004 (2)	0.0005 (19)	−0.003 (2)
C(6P)	0.014 (2)	0.041 (3)	0.016 (2)	−0.004 (2)	0.0004 (17)	0.003 (2)
C(7C)	0.086 (7)	0.110 (9)	0.054 (5)	−0.016 (6)	0.010 (4)	0.005 (5)

### Geometric parameters (Å, °)

**Table:**

W(1)—C(5)	2.162 (5)	N(3P)—C(2P)	1.328 (6)
W(1)—C(8)	2.146 (4)	N(3)—C(3)	1.145 (6)
W(1)—C(6)	2.153 (4)	N(7)—C(7)	1.141 (6)
W(1)—C(1)	2.149 (4)	N(1P)—C(2P)	1.339 (6)
W(1)—C(3)	2.153 (4)	N(1P)—C(6P)	1.341 (6)
W(1)—C(2)	2.170 (4)	N(1C)—C(6C)	1.331 (7)
W(1)—C(7)	2.173 (4)	N(1C)—C(2C)	1.322 (6)
W(1)—C(4)	2.174 (4)	N(2)—C(2)	1.145 (6)
Cu(1)—O(1)	1.964 (3)	N(8C)—C(7C)	1.202 (12)
Cu(1)—O(1)^i^	1.964 (3)	N(1)—C(1)	1.141 (6)
Cu(1)—N(1P)	2.086 (3)	C(4P)—C(5P)	1.381 (6)
Cu(1)—N(1P)^i^	2.086 (3)	C(5P)—C(6P)	1.383 (7)
Cu(1)—N(1)	2.368 (3)	C(6C)—C(5C)	1.364 (8)
Cu(1)—N(1)^i^	2.368 (3)	C(5C)—C(4C)	1.380 (11)
Cu(2)—N(8)^ii^	2.301 (4)	C(4C)—C(3C)	1.368 (12)
Cu(2)—N(6)^iii^	1.975 (3)	C(4C)—C(7C)	1.465 (11)
Cu(2)—N(3P)	2.076 (4)	C(3C)—C(2C)	1.386 (7)
Cu(2)—N(3)^iv^	1.990 (3)	C(4P)—H(4P)	0.958
Cu(2)—N(1C)	2.046 (3)	C(5P)—H(5P)	0.958
Cu(2)—N(2)	2.399 (4)	C(2P)—H(2P)	0.955
N(8)—C(8)	1.154 (6)	C(6C)—H(6C)	0.965
N(5)—C(5)	1.149 (7)	C(5C)—H(5C)	0.967
N(4)—C(4)	1.143 (6)	C(3C)—H(3C)	0.965
N(6)—C(6)	1.157 (6)	C(2C)—H(2C)	0.964
N(3P)—C(4P)	1.340 (6)	C(6P)—H(6P)	0.960
			
O(1)···O(2W)	2.615 (5)	N(8C)···C(3)^vii^	3.564 (10)
O(1)···N(4)^v^	2.770 (5)	N(8C)···C(6C)^vii^	3.498 (12)
O(2W)···O(1)	2.615 (5)	C(3)···N(8C)^vii^	3.564 (10)
O(2W)···N(5)^i^	2.901 (6)	C(2P)···O(2W)	3.367 (6)
O(2W)···N(7)^iii^	2.849 (5)	C(6C)···N(8C)^vii^	3.498 (12)
O(2W)···N(1P)	3.090 (6)	C(5C)···O(2W)^x^	3.472 (8)
O(2W)···C(2P)	3.367 (6)	C(5C)···N(5)^ix^	3.326 (9)
O(2W)···C(5C)^ii^	3.472 (8)	C(5C)···C(7C)^vii^	3.546 (13)
O(2W)···C(6P)	3.268 (7)	C(4C)···C(4C)^vii^	3.559 (9)
N(5)···O(2W)^i^	2.901 (6)	C(6P)···O(2W)	3.268 (7)
N(5)···N(8C)^vi^	3.318 (13)	C(7C)···N(5)^ix^	3.301 (14)
N(5)···C(5C)^vi^	3.326 (9)	C(7C)···C(5C)^vii^	3.546 (13)
N(5)···C(7C)^vi^	3.301 (14)	O(2W)···H(5C)^ii^	2.705
N(4)···O(1)^v^	2.770 (5)	O(2W)···H(3C)^xi^	3.087
N(3)···N(8C)^vii^	3.398 (10)	O(2W)···H(6P)	3.578
N(7)···O(2W)^viii^	2.849 (5)	N(5)···H(5C)^vi^	2.510
N(7)···N(8C)^vii^	3.462 (13)	N(7)···H(3C)^vii^	3.207
N(1P)···O(2W)	3.090 (6)	C(6P)···H(6P)^xii^	3.304
N(1C)···N(8C)^vii^	3.513 (11)	H(5C)···O(2W)^x^	2.705
N(2)···N(8C)^vii^	3.563 (10)	H(5C)···N(5)^ix^	2.510
N(8C)···N(5)^ix^	3.318 (13)	H(3C)···O(2W)^xiii^	3.087
N(8C)···N(3)^vii^	3.398 (10)	H(3C)···N(7)^vii^	3.207
N(8C)···N(7)^vii^	3.462 (13)	H(6P)···O(2W)	3.578
N(8C)···N(1C)^vii^	3.513 (11)	H(6P)···C(6P)^xii^	3.304
N(8C)···N(2)^vii^	3.563 (10)	H(6P)···H(6P)^xii^	3.136
			
C(5)—W(1)—C(8)	70.83 (18)	N(3P)—Cu(2)—N(2)	91.72 (14)
C(5)—W(1)—C(6)	79.62 (19)	N(3)^iv^—Cu(2)—N(1C)	90.59 (16)
C(5)—W(1)—C(1)	75.05 (19)	N(3)^iv^—Cu(2)—N(2)	87.51 (14)
C(5)—W(1)—C(3)	142.29 (18)	N(1C)—Cu(2)—N(2)	90.00 (15)
C(5)—W(1)—C(2)	119.52 (18)	Cu(2)^x^—N(8)—C(8)	171.0 (3)
C(5)—W(1)—C(7)	139.15 (19)	Cu(2)^viii^—N(6)—C(6)	165.0 (3)
C(5)—W(1)—C(4)	73.28 (18)	Cu(2)—N(3P)—C(4P)	120.1 (3)
C(8)—W(1)—C(6)	82.92 (17)	Cu(2)—N(3P)—C(2P)	121.8 (3)
C(8)—W(1)—C(1)	110.23 (17)	C(4P)—N(3P)—C(2P)	117.6 (4)
C(8)—W(1)—C(3)	146.68 (16)	Cu(2)^xiv^—N(3)—C(3)	161.7 (3)
C(8)—W(1)—C(2)	76.94 (17)	Cu(1)—N(1P)—C(2P)	121.9 (3)
C(8)—W(1)—C(7)	76.43 (17)	Cu(1)—N(1P)—C(6P)	120.7 (3)
C(8)—W(1)—C(4)	140.49 (17)	C(2P)—N(1P)—C(6P)	117.4 (4)
C(6)—W(1)—C(1)	145.12 (17)	Cu(2)—N(1C)—C(6C)	119.7 (3)
C(6)—W(1)—C(3)	103.42 (17)	Cu(2)—N(1C)—C(2C)	121.7 (3)
C(6)—W(1)—C(2)	144.47 (17)	C(6C)—N(1C)—C(2C)	118.5 (4)
C(6)—W(1)—C(7)	72.62 (17)	Cu(2)—N(2)—C(2)	168.1 (3)
C(6)—W(1)—C(4)	74.85 (17)	Cu(1)—N(1)—C(1)	149.5 (3)
C(1)—W(1)—C(3)	83.36 (17)	W(1)—C(5)—N(5)	178.7 (5)
C(1)—W(1)—C(2)	70.17 (17)	W(1)—C(8)—N(8)	177.4 (4)
C(1)—W(1)—C(7)	140.87 (18)	W(1)—C(6)—N(6)	177.4 (3)
C(1)—W(1)—C(4)	75.16 (17)	W(1)—C(1)—N(1)	177.8 (4)
C(3)—W(1)—C(2)	79.79 (16)	W(1)—C(3)—N(3)	175.2 (3)
C(3)—W(1)—C(7)	74.54 (17)	W(1)—C(2)—N(2)	176.2 (4)
C(3)—W(1)—C(4)	71.47 (16)	W(1)—C(7)—N(7)	178.4 (4)
C(2)—W(1)—C(7)	74.36 (17)	W(1)—C(4)—N(4)	177.8 (4)
C(2)—W(1)—C(4)	136.94 (17)	N(3P)—C(4P)—C(5P)	121.5 (4)
C(7)—W(1)—C(4)	124.99 (16)	C(4P)—C(5P)—C(6P)	117.4 (4)
O(1)—Cu(1)—O(1)^i^	180.00 (18)	N(3P)—C(2P)—N(1P)	124.8 (4)
O(1)—Cu(1)—N(1P)	90.05 (15)	N(1C)—C(6C)—C(5C)	123.3 (5)
O(1)—Cu(1)—N(1P)^i^	89.95 (15)	C(6C)—C(5C)—C(4C)	117.4 (6)
O(1)—Cu(1)—N(1)	91.15 (14)	C(5C)—C(4C)—C(3C)	120.7 (6)
O(1)—Cu(1)—N(1)^i^	88.85 (14)	C(5C)—C(4C)—C(7C)	127.1 (8)
O(1)^i^—Cu(1)—N(1P)	89.95 (15)	C(3C)—C(4C)—C(7C)	112.1 (8)
O(1)^i^—Cu(1)—N(1P)^i^	90.05 (15)	C(4C)—C(3C)—C(2C)	117.3 (6)
O(1)^i^—Cu(1)—N(1)	88.85 (14)	N(1C)—C(2C)—C(3C)	122.7 (5)
O(1)^i^—Cu(1)—N(1)^i^	91.15 (14)	N(1P)—C(6P)—C(5P)	121.3 (4)
N(1P)—Cu(1)—N(1P)^i^	180.0 (2)	N(8C)—C(7C)—C(4C)	173.5 (12)
N(1P)—Cu(1)—N(1)	89.52 (14)	N(3P)—C(4P)—H(4P)	119.1
N(1P)—Cu(1)—N(1)^i^	90.48 (14)	C(5P)—C(4P)—H(4P)	119.5
N(1P)^i^—Cu(1)—N(1)	90.48 (14)	C(4P)—C(5P)—H(5P)	121.4
N(1P)^i^—Cu(1)—N(1)^i^	89.52 (14)	C(6P)—C(5P)—H(5P)	121.2
N(1)—Cu(1)—N(1)^i^	180.0 (2)	N(3P)—C(2P)—H(2P)	117.6
N(8)^ii^—Cu(2)—N(6)^iii^	87.57 (15)	N(1P)—C(2P)—H(2P)	117.6
N(8)^ii^—Cu(2)—N(3P)	88.40 (15)	N(1C)—C(6C)—H(6C)	118.1
N(8)^ii^—Cu(2)—N(3)^iv^	93.94 (15)	C(5C)—C(6C)—H(6C)	118.6
N(8)^ii^—Cu(2)—N(1C)	89.99 (15)	C(6C)—C(5C)—H(5C)	121.2
N(8)^ii^—Cu(2)—N(2)	178.55 (14)	C(4C)—C(5C)—H(5C)	121.4
N(6)^iii^—Cu(2)—N(3P)	92.69 (15)	C(4C)—C(3C)—H(3C)	121.2
N(6)^iii^—Cu(2)—N(3)^iv^	177.57 (16)	C(2C)—C(3C)—H(3C)	121.5
N(6)^iii^—Cu(2)—N(1C)	91.31 (16)	N(1C)—C(2C)—H(2C)	118.3
N(6)^iii^—Cu(2)—N(2)	90.98 (15)	C(3C)—C(2C)—H(2C)	119.0
N(3P)—Cu(2)—N(3)^iv^	85.46 (15)	N(1P)—C(6P)—H(6P)	119.2
N(3P)—Cu(2)—N(1C)	175.62 (15)	C(5P)—C(6P)—H(6P)	119.5
			
C(5)—W(1)—C(8)—N(8)	63 (8)	N(1P)^i^—Cu(1)—N(1)—C(1)	−91.9 (7)
C(8)—W(1)—C(5)—N(5)	105 (22)	N(1)—Cu(1)—N(1P)^i^—C(2P)^i^	177.7 (3)
C(5)—W(1)—C(6)—N(6)	75 (9)	N(1)—Cu(1)—N(1P)^i^—C(6P)^i^	−3.0 (4)
C(6)—W(1)—C(5)—N(5)	−169 (18)	N(1P)^i^—Cu(1)—N(1)^i^—C(1)^i^	−88.1 (7)
C(5)—W(1)—C(1)—N(1)	34 (10)	N(1)^i^—Cu(1)—N(1P)^i^—C(2P)^i^	−2.3 (3)
C(1)—W(1)—C(5)—N(5)	−13 (21)	N(1)^i^—Cu(1)—N(1P)^i^—C(6P)^i^	177.0 (4)
C(5)—W(1)—C(3)—N(3)	−14 (4)	N(8)^ii^—Cu(2)—N(6)^iii^—C(6)^iii^	22.8 (14)
C(3)—W(1)—C(5)—N(5)	−70 (22)	N(6)^iii^—Cu(2)—N(8)^ii^—C(8)^ii^	162 (2)
C(5)—W(1)—C(2)—N(2)	−68 (6)	N(8)^ii^—Cu(2)—N(3P)—C(4P)	−30.3 (3)
C(2)—W(1)—C(5)—N(5)	43 (22)	N(8)^ii^—Cu(2)—N(3P)—C(2P)	157.6 (3)
C(5)—W(1)—C(7)—N(7)	132 (16)	N(3P)—Cu(2)—N(8)^ii^—C(8)^ii^	70 (2)
C(7)—W(1)—C(5)—N(5)	144 (22)	N(8)^ii^—Cu(2)—N(3)^iv^—C(3)^iv^	131.3 (12)
C(5)—W(1)—C(4)—N(4)	64 (11)	N(3)^iv^—Cu(2)—N(8)^ii^—C(8)^ii^	−16 (2)
C(4)—W(1)—C(5)—N(5)	−92 (22)	N(8)^ii^—Cu(2)—N(1C)—C(6C)	141.8 (4)
C(8)—W(1)—C(6)—N(6)	147 (9)	N(8)^ii^—Cu(2)—N(1C)—C(2C)	−35.7 (3)
C(6)—W(1)—C(8)—N(8)	−19 (8)	N(1C)—Cu(2)—N(8)^ii^—C(8)^ii^	−106 (2)
C(8)—W(1)—C(1)—N(1)	−29 (10)	N(8)^ii^—Cu(2)—N(2)—C(2)	−42 (6)
C(1)—W(1)—C(8)—N(8)	128 (8)	N(2)—Cu(2)—N(8)^ii^—C(8)^ii^	164 (4)
C(8)—W(1)—C(3)—N(3)	174 (4)	N(6)^iii^—Cu(2)—N(3P)—C(4P)	−117.8 (4)
C(3)—W(1)—C(8)—N(8)	−123 (8)	N(6)^iii^—Cu(2)—N(3P)—C(2P)	70.2 (3)
C(8)—W(1)—C(2)—N(2)	−127 (6)	N(3P)—Cu(2)—N(6)^iii^—C(6)^iii^	111.1 (14)
C(2)—W(1)—C(8)—N(8)	−169 (8)	N(6)^iii^—Cu(2)—N(3)^iv^—C(3)^iv^	3(4)
C(8)—W(1)—C(7)—N(7)	169 (16)	N(3)^iv^—Cu(2)—N(6)^iii^—C(6)^iii^	151 (3)
C(7)—W(1)—C(8)—N(8)	−93 (8)	N(6)^iii^—Cu(2)—N(1C)—C(6C)	−130.7 (4)
C(8)—W(1)—C(4)—N(4)	89 (11)	N(6)^iii^—Cu(2)—N(1C)—C(2C)	51.9 (3)
C(4)—W(1)—C(8)—N(8)	37 (8)	N(1C)—Cu(2)—N(6)^iii^—C(6)^iii^	−67.1 (14)
C(6)—W(1)—C(1)—N(1)	78 (10)	N(6)^iii^—Cu(2)—N(2)—C(2)	−40.2 (17)
C(1)—W(1)—C(6)—N(6)	31 (9)	N(2)—Cu(2)—N(6)^iii^—C(6)^iii^	−157.2 (14)
C(6)—W(1)—C(3)—N(3)	76 (4)	N(3P)—Cu(2)—N(3)^iv^—C(3)^iv^	43.2 (12)
C(3)—W(1)—C(6)—N(6)	−66 (9)	N(3)^iv^—Cu(2)—N(3P)—C(4P)	63.8 (3)
C(6)—W(1)—C(2)—N(2)	176 (5)	N(3)^iv^—Cu(2)—N(3P)—C(2P)	−108.3 (3)
C(2)—W(1)—C(6)—N(6)	−158 (9)	N(3P)—Cu(2)—N(1C)—C(6C)	73 (2)
C(6)—W(1)—C(7)—N(7)	82 (16)	N(3P)—Cu(2)—N(1C)—C(2C)	−104 (2)
C(7)—W(1)—C(6)—N(6)	−135 (9)	N(1C)—Cu(2)—N(3P)—C(4P)	38 (2)
C(6)—W(1)—C(4)—N(4)	147 (11)	N(1C)—Cu(2)—N(3P)—C(2P)	−134 (2)
C(4)—W(1)—C(6)—N(6)	−0(8)	N(3P)—Cu(2)—N(2)—C(2)	52.5 (17)
C(1)—W(1)—C(3)—N(3)	−69 (4)	N(2)—Cu(2)—N(3P)—C(4P)	151.1 (3)
C(3)—W(1)—C(1)—N(1)	−178 (9)	N(2)—Cu(2)—N(3P)—C(2P)	−20.9 (3)
C(1)—W(1)—C(2)—N(2)	−10 (6)	N(3)^iv^—Cu(2)—N(1C)—C(6C)	47.8 (4)
C(2)—W(1)—C(1)—N(1)	−96 (10)	N(3)^iv^—Cu(2)—N(1C)—C(2C)	−129.6 (3)
C(1)—W(1)—C(7)—N(7)	−86 (16)	N(1C)—Cu(2)—N(3)^iv^—C(3)^iv^	−138.7 (12)
C(7)—W(1)—C(1)—N(1)	−122 (10)	N(3)^iv^—Cu(2)—N(2)—C(2)	137.8 (17)
C(1)—W(1)—C(4)—N(4)	−15 (11)	N(2)—Cu(2)—N(3)^iv^—C(3)^iv^	−48.7 (12)
C(4)—W(1)—C(1)—N(1)	110 (10)	N(1C)—Cu(2)—N(2)—C(2)	−131.6 (17)
C(3)—W(1)—C(2)—N(2)	77 (6)	N(2)—Cu(2)—N(1C)—C(6C)	−39.7 (3)
C(2)—W(1)—C(3)—N(3)	−140 (4)	N(2)—Cu(2)—N(1C)—C(2C)	142.9 (3)
C(3)—W(1)—C(7)—N(7)	−28 (16)	Cu(2)^x^—N(8)—C(8)—W(1)	29 (10)
C(7)—W(1)—C(3)—N(3)	144 (4)	Cu(2)^viii^—N(6)—C(6)—W(1)	8(10)
C(3)—W(1)—C(4)—N(4)	−103 (11)	Cu(2)—N(3P)—C(4P)—C(5P)	−171.8 (4)
C(4)—W(1)—C(3)—N(3)	8(4)	Cu(2)—N(3P)—C(2P)—N(1P)	170.1 (3)
C(2)—W(1)—C(7)—N(7)	−111 (16)	C(4P)—N(3P)—C(2P)—N(1P)	−2.1 (7)
C(7)—W(1)—C(2)—N(2)	154 (6)	C(2P)—N(3P)—C(4P)—C(5P)	0.6 (7)
C(2)—W(1)—C(4)—N(4)	−52 (11)	Cu(2)^xiv^—N(3)—C(3)—W(1)	−3(5)
C(4)—W(1)—C(2)—N(2)	29 (6)	Cu(1)—N(1P)—C(2P)—N(3P)	−177.2 (3)
C(7)—W(1)—C(4)—N(4)	−157 (11)	Cu(1)—N(1P)—C(6P)—C(5P)	178.7 (4)
C(4)—W(1)—C(7)—N(7)	26 (16)	C(2P)—N(1P)—C(6P)—C(5P)	−0.6 (8)
O(1)—Cu(1)—N(1P)—C(2P)	−88.9 (3)	C(6P)—N(1P)—C(2P)—N(3P)	2.1 (7)
O(1)—Cu(1)—N(1P)—C(6P)	91.8 (4)	Cu(2)—N(1C)—C(6C)—C(5C)	−175.8 (5)
O(1)—Cu(1)—N(1P)^i^—C(2P)^i^	−91.1 (3)	Cu(2)—N(1C)—C(2C)—C(3C)	175.9 (4)
O(1)—Cu(1)—N(1P)^i^—C(6P)^i^	88.2 (4)	C(6C)—N(1C)—C(2C)—C(3C)	−1.6 (7)
O(1)—Cu(1)—N(1)—C(1)	178.2 (7)	C(2C)—N(1C)—C(6C)—C(5C)	1.7 (8)
O(1)—Cu(1)—N(1)^i^—C(1)^i^	1.8 (7)	Cu(2)—N(2)—C(2)—W(1)	−20 (7)
O(1)^i^—Cu(1)—N(1P)—C(2P)	91.1 (3)	Cu(1)—N(1)—C(1)—W(1)	9(10)
O(1)^i^—Cu(1)—N(1P)—C(6P)	−88.2 (4)	N(3P)—C(4P)—C(5P)—C(6P)	0.8 (8)
O(1)^i^—Cu(1)—N(1P)^i^—C(2P)^i^	88.9 (3)	C(4P)—C(5P)—C(6P)—N(1P)	−0.7 (8)
O(1)^i^—Cu(1)—N(1P)^i^—C(6P)^i^	−91.8 (4)	N(1C)—C(6C)—C(5C)—C(4C)	−0.8 (9)
O(1)^i^—Cu(1)—N(1)—C(1)	−1.8 (7)	C(6C)—C(5C)—C(4C)—C(3C)	−0.3 (7)
O(1)^i^—Cu(1)—N(1)^i^—C(1)^i^	−178.2 (7)	C(6C)—C(5C)—C(4C)—C(7C)	−176.1 (7)
N(1P)—Cu(1)—N(1)—C(1)	88.1 (7)	C(5C)—C(4C)—C(3C)—C(2C)	0.3 (7)
N(1)—Cu(1)—N(1P)—C(2P)	2.3 (3)	C(5C)—C(4C)—C(7C)—N(8C)	−22 (10)
N(1)—Cu(1)—N(1P)—C(6P)	−177.0 (4)	C(3C)—C(4C)—C(7C)—N(8C)	161 (9)
N(1P)—Cu(1)—N(1)^i^—C(1)^i^	91.9 (7)	C(7C)—C(4C)—C(3C)—C(2C)	176.8 (6)
N(1)^i^—Cu(1)—N(1P)—C(2P)	−177.7 (3)	C(4C)—C(3C)—C(2C)—N(1C)	0.6 (8)
N(1)^i^—Cu(1)—N(1P)—C(6P)	3.0 (4)		

Symmetry codes: (i) −*x*+1, −*y*+1, −*z*+1; (ii) −*x*+1/2, *y*−1/2, −*z*+1/2; (iii) −*x*+3/2, *y*−1/2, −*z*+1/2; (iv) *x*−1, *y*, *z*; (v) −*x*+2, −*y*+1, −*z*+1; (vi) *x*+1/2, −*y*+3/2, *z*+1/2; (vii) −*x*+1, −*y*+1, −*z*; (viii) −*x*+3/2, *y*+1/2, −*z*+1/2; (ix) *x*−1/2, −*y*+3/2, *z*−1/2; (x) −*x*+1/2, *y*+1/2, −*z*+1/2; (xi) *x*+1/2, −*y*+1/2, *z*+1/2; (xii) −*x*, −*y*+1, −*z*+1; (xiii) *x*−1/2, −*y*+1/2, *z*−1/2; (xiv) *x*+1, *y*, *z*.
